# Mental Health and Psychosocial Support During COVID-19: A Review of Health Guidelines in Sub-Saharan Africa

**DOI:** 10.3389/fpsyt.2021.571342

**Published:** 2021-05-20

**Authors:** Keneilwe Molebatsi, Otsetswe Musindo, Vuyokazi Ntlantsana, Grace Nduku Wambua

**Affiliations:** ^1^Department of Psychiatry, Faculty of Medicine, University of Botswana, Gaborone, Botswana; ^2^Department of Psychiatry, Nelson Mandela School of Clinical Medicine, University of Kwa-Zulu Natal, Durban, South Africa; ^3^Selibe Phikwe Government Hospital, Ministry of Health and Wellness, Selibe Phikwe, Botswana; ^4^Department of Clinical, Neuro and Developmental Psychology, Amsterdam Public Health Research Institute, Vrije Universiteit Amsterdam, Amsterdam, Netherlands

**Keywords:** Sub-Saharan Africa, mental health service, COVID-19, mental health guidelines, prevention policies

## Abstract

The COVID-19 pandemic brought in its wake an unforeseen mental health crisis. The World Health Organization published a guideline as a way of supporting mental health and psychosocial well-being of different groups during this pandemic. The impact of the pandemic has pushed governments to put measures in place to curb not only the physical health of individuals but their mental health and psychosocial well-being as well. The aim of our paper was to review mental health guidelines of some Sub Saharan African (SSA) countries: (i) to assess their appropriateness for the immediate mental health needs at this time, (ii) to form as a basis for ongoing reflection as the current pandemic evolves. Guidelines were retrieved openly from internet search and some were requested from mental health practitioners in various SSA countries. The authors designed a semi structured questionnaire, as a self-interview guide to gain insight on the experience of COVID-19 from experts in the mental health sector in the various countries. While we used a document analysis approach to analyze the data, we made use of the Mental Health Preparedness and Action Framework to discuss our findings. We received health or mental health guidelines from 10 SSA countries. Cameroon, Kenya, South Africa, Tanzania, and Uganda all had mental health guidelines or mental health component in their health guidelines. Our experts highlight that the mental health needs of the people are of concern during this pandemic but have not been given priority. They go further to suggest that the mental health needs are slightly different during this time and requiring a different approach especially considering the measures taken to curb the spread of disease. We conclude that despite the provision of Mental Health and Psychosocial Support guidelines, gaps still exist making them inadequate to meet the mental health needs of their communities.

## Introduction

The Severe Acute Respiratory Syndrome Coronavirus 2 (COVID-19) which emerged in December 2019 in the city of Wuhan, China has dominated headlines, spread globally, and resulted in a pandemic (1). To prevent transmission of the COVID-19 disease, countries responded by restricting movements. Lockdowns or quarantines have not only impacted the global economy and day-to-day lives, it has also caused a parallel pandemic of fear to the local and global community (2).

The entrance of COVID-19 into Sub-Saharan Africa (SSA) was later than most, with the first identified case in Nigeria in February 2020 (3). The effects of the disease have not been as severe as seen in western countries such as Italy and the United States of America (4). Africa is not a stranger to pandemics with Ebola, Yellow fever, Chikungunya virus and Zika virus among the recent culprits (5, 6), which has enabled countries to create or empower national public health institutes to focus on disease preparedness and responsiveness in the case of a pandemic (7). The mental health crisis which results from the multi-level impact of the pandemic has however gone on unattended (8). The contagion may, at biological level, directly affect the central nervous system thereby causing neuropsychiatric manifestations, at psychological level the impact of morbidity and mortality exceeds even the most resilient of societies (8, 9). At societal level, the opportunity cost of the pandemic is yet to be determined. There is a looming crisis on children's health where with the threat to non-communicable diseases such as malnutrition, and the threat to sustained immunization exists (10). In addition, the economic costs of the pandemic are high, particularly for the vulnerable group of low-income urban informal settlement dwellers who live under conditions of economic constraints who are put under further pressure by lockdown rules (11). The mental health impact of the additional burden of social distancing requirements in situations of crowded living circumstances, and the difficult choice of risking infection or going hungry needs consideration.

Significant impact on the well-being of those affected, their family, community members as well as health care workers have been highlighted (2). Emerging research from across the globe demonstrates that fear may lead to anxiety and depression for example, research from China indicated that 8.4% of the general population reported severe to extremely severe anxiety while 4.3% reported severe to extremely severe depression (12). Extreme states of anxiety, stresses, social stigma, and discrimination are associated with COVID-19 hence there is need of enhancing mental and psychosocial well-being of people affected. To mitigate the risk of negative psychological outcomes caused by COVID-19, the World Health Organization (WHO) department of Mental Health and Substance Use published a document, “*Mental health and psychosocial consideration during COVID-19 outbreak.”* It was a way of supporting mental health and psychosocial well-being of different population groups during this pandemic (13). This document provides information to strengthen preparedness and response plans with regards to mental health and psychosocial consequences of COVID-19 outbreak.

Impacts of pandemics such as COVID-19 have highlighted the need for governments to put measures in place to curb not only the physical health of individuals but their mental and psychosocial well-being as well. In this paper, we review mental health guidelines of some Sub-Saharan African countries to achieve the following objectives:

- To assess the appropriateness of the guidelines in our context
- To serve as information sharing to allow for ongoing dialogue across mental health sectors on the African continent
- To form as a basis for ongoing reflection as this current pandemic evolves.

## Conceptual Framework

We made use of the *Mental Health Preparedness and Action Framework (MHPAF)* developed by Ransing et al. (14) which we adapted for our paper. They describe five interlinked components and suggest that inadequate preparation of one component has a ripple effect and can affect the success of mental health interventions before, during and after a pandemic.

1. *Preparation and coordination:* Ransing et al. (14) suggest that in the early phase of a pandemic, this component should be directed toward preparing infrastructure. These include for example Mental Health Surveillance System (MHSS) that enables systematic data collection, analysis, interpretation, and the timely dissemination of the data to those responsible for prevention and control of the epidemic; training of volunteers and health care workers in psychological first aid (PFA) materials, designation of special clinics for mental health (also a shift to telepsychiatry).
2. *Monitoring and Assessment:* The MHPAF recommends that the MHSS team should prepare a written pandemic mental health emergency plan with special attention to the population at risk. The use of alternative forms of technology such as telepsychiatry, digital platforms, dedicated hotlines and mental health applications can be used for assessment and monitoring of mental health and have the potential to reduce the treatment gap. MHSS should be equipped with such tools for monitoring and assessment in all phases of the pandemic.
3. *Communication* is a key component in the mental health response during the pandemic with previous reports highlighting the need for up-to-date information on an ongoing basis throughout the period. The presentation of information by trusted public health officials helps in minimizing fear and hysteria (14). Reducing the mental distress due to misinformation and “myths” is also important in the early phase of the pandemic. Media (both mainstream and social) are the most frequently used approaches by the government to address misinformation. It is suggested that the MHSS team or public health system should be well-equipped for continuous monitoring and should address myths promptly.
4. *Sustainability of mental health care services –* In this we look at the availability of resources—human and financial—that are critical for strengthening the country's preparedness and response to a pandemic. Are funds being directed to MH response? Are the specialist services adequately trained to meet the needs of pandemic, availability? Coordination and collaboration has also been sighted as important because a lack of coordination and collaboration within the healthcare system can affect the delivery of mental health services during a pandemic.

## Methodology

We searched online for health guidelines for the management of COVID-19. We also requested guidelines from mental health practitioners in various SSA countries. Emails were sent to a selected number of mental health practitioners in SSA countries and those who responded to a second reminder were included in the study. We received guidelines from Botswana, Cameroon, Ethiopia, Kenya, Republic of South Africa (South Africa), Sierra Leone, Tanzania, Uganda, Zambia, and Zimbabwe. Ethical approval was not sought for this study as research on publicly available data does not require an ethical review (15).

The authors developed a semi structured questionnaire which was used as a self-interview guide to gain an understanding of perceptions of mental health practitioners/clinicians in various SSA countries regarding their country's mental health status and response during the pandemic (16, 17). Examples of the questions include “*How would you describe the situation the country with regards to mental health during this COVID-19 crisis*?,” “*what are some of the mental health needs of the country at this time?*,” “*In your opinion, what are some of the mental health needs of the country at this time?*,” “*are issues of mental health a priority during this crisis? (availability of services, resources human, and financial).”*

The study used a document analysis methodology to assess MHPSS guidelines and recommendations developed by the various countries in the wake of COVID-19 (18). Document analysis is often used with other qualitative methods to allow for triangulation information which attempts to provide a convergence of evidence. The authors believe this was necessary to serve as input into present management, future policy guidelines and strategic planning processes for future epidemics/pandemics in SSA. Authors KM, OM, and GNW reviewed the various guidelines and responses from the online qualitative survey. Content analysis was carried out and the data was coded into broad themes, guided by our objectives.

## Results

We received health and mental health policy guidelines from 10 countries, and we were able to interview 10 experts from these countries (3 clinical psychologists, 1 mental health clinician and researcher, 6 psychiatrists). We highlight country specific COVID-19 demographics as at 9th June 2020 in Table 1 (19). *Five* countries: Cameroon, Kenya (20), South Africa (21), Tanzania (22), and Uganda (23) all had mental health guidelines or a mental health component in their health guidelines. In Table 2 we summarize the mental health guidelines developed between March to April 2020 as a response to the COVID-19 pandemic outbreak in SSA. Other countries including, Botswana, Sierra Leone, Ethiopia, Zambia, and Zimbabwe have health guidelines or Standard Operating Procedures (SOPs) for the management of the COVID-19, but these guidelines did not include Mental Health or Psychosocial support.

**Table 1:** COVID-19 Country demographics as at 9th June 2020 (19).

	**First case**	**Confirmed cases**	**Dead**	**Recovered**	**Measures taken by country to curb spread of disease**
Kenya	March 13th	2,862	85	849	Lockdown, curfew (7 P.M.−5 A.M.), travel ban between counties and abroad, mandatory quarantine for those coming in from abroad.
Republic of South Africa	March 5th	50,879	1,080	26,099	A travel ban between high risk countries 13 days after the first case. Followed by a total lockdown which began 26th March, for 5 weeks, followed by a phased easing of lockdown regulations.
Tanzania	March 16th	509	21	183	No restrictions.
Uganda	March 21st	657		118	Restricted movements except essential travel, closure of non-essential shopping outlets, curfew (7 P.M.−6:30 A.M.).
Cameroon	March 6th	8,312	212	4,794	Schools closure, mandatory masks, restricted external travel.
Sierra Leone	March 31st	1,001	49	611	Declared a 3-day nationwide lockdown covering the period Sunday, 3rd May to Tuesday, 5th of May 2020, afterwards inter-district travel restricted.
Botswana	March 30th	42	1	24	Lockdown declared on 02 April 2020 for 28 days followed by phased lifting of the lockdown restriction for 3 weeks. mandatory quarantine for those coming from high risk countries
Zambia	March 18th	1,200	10	912	Partial lockdown form 20 March 2020: movement not restricted but the population encouraged to stay home, schools and alcohol selling outlets closed.
Zimbabwe	March 21st	287	4	46	National Lockdown and prohibition of gatherings was legislated for the 21 day period commending 30 March 2020, restricting movement of the public with the exception to those providing essential services. Mandatory 21-day quarantine for those returning from affected countries

**Table 2:** Summary of guidelines and SOP's of MH and PSS during the COVID-19 outbreak in South Africa, Kenya, Uganda, Tanzania, Cameroon between March and April 2020.

	**Kenya**	**RSA**	**Cameroon**	**Uganda**	**Tanzania**
**Mental health guideline/standard operating procedures**	**April, 2020**	**April, 2020**	**April, 2020**	**April, 2020**	**April, 2020**
Objective	Yes	Yes	No	Yes	Yes
	- To aid in provision of quality and effective screening, management and provision of mental health and psychosocial support to people suspected or diagnosed to have COVID-19.	- To provide information to promote and protect the mental well-being of the population and to raise awareness about MH problems that may arise due to the COVID-19 outbreak. - To direct head of health establishments, health service providers and informal caregivers on actions to be taken to identify and manage MH problems that may arise during this time. - To ensure that psychiatric facilities comply with measures that have already been prescribed to manage the COVID-19 outbreak.		- To facilitate the contribution of a sense of normalcy, foster the healing process and enhances resilience of the affected populations through the support and management of stress, to prevent the negative psychological outcomes including anxiety, depression, panic attacks, and sleep disturbances.	- To provide guidance on implementation of psychosocial support activities during and after COVID outbreaks.
Focus populations	- To cover the needs of the population, - People in treatment for COVID-19, those in quarantine and isolation, - People with mental health conditions requiring continuing care in these settings, - Health workers	- All populations - Provide support to affected individuals, families, and staff in need. - Primary health care workers	- People in isolation (patients) or in isolation (suspected of infection) - Relatives of people in isolation and/or quarantine, including children - The (para) medical staff in charge of people directly or indirectly infected with the infection	- Healthcare workers, health facility managers, - Care providers of children, - Older adults, - People with underlying health conditions, - People in isolation	- Individual, families, health workers and community - Patients confirmed of COVID-19 in treatment/isolation facilities/self-solation, - Contacts in self-isolation, - Individuals in mandatory self-isolation at home/quarantine facility, - Children affected by COVID-19
Interventions	- Psychological first aid - Enhancing coping skills	- N/A (see note below)	- Psychological first aid - Stress management - Enhancing coping skills	- Psychological first aid - Enhancing coping skills	- Psychological first aid - Enhancing coping skills
Who is assisting in provision of service	- Clinicians, health care workers, counselors, and psychologists	- Multi-disciplinary mental health specialists' team comprised of a psychiatrist, psychologist, nurse, occupational therapist and social worker	- Not identified	- Not identified	- Psychosocial team is composed of social workers, social welfare officers, clinical and community psychologists, risk communication and health promotion experts, charity social care organization representatives, community development officers and psychiatric medic such as clinical officer and nurses. - *The guideline also outlines their role during and after the outbreak*.
Was it developed collaboratively?	- Yes	- Not mentioned	- Not mentioned	- Yes	- Not mentioned
In line with WHO guidelines	- Yes	- No	- Yes (some and some)	- Yes	- Yes

Mental health experts suggest that mental health of the communities is of concern during this pandemic. Anxiety heightened by the fear of contracting the virus and uncertainties due to poor information coming from governments and media. Extended lockdowns, curfews, and loss of work opportunities are impacting the economic livelihoods of many thus influencing their mental health. The potential rise in persons needing mental health services was also an issue of worry as many countries lack the human resource to cater to increased need of persons needing care.

*The situation is a combination of variety of issues ranging from anxiety due to the uncertainty of clear information about the virus itself and panic due to the unknown preparedness in containing the spread and fatalities should they happen*. (Kenya)

*Mostly anxiety brought about fear of the unknown and uncertainty regarding when this will be over, will people still have their jobs, how many people are we going to lose to this disease etc. Depression tends to co-exist hand in hand with anxiety and this situation is no different. Disruption of daily routines, confinement, fear of unemployment, stopping income generating activities for the informal sector all contributory*. (Botswana)

*Our numbers are raising we are yet to reach the peak of the pandemic. …We are witnessing lots of fear worry and COVID induced anxiety among the population. The Lockdown and curfew are making many people restless due to loss of power to earn and creating lots of separation anxiety especially to family members locked away from their loved ones*. (Kenya)

*The health system has suddenly come under an additional strain of COVID with COVID patients taking priority over mental health conditions, patients who would benefit from inpatient care have to be treated as outpatients to make room for the more urgent COVID cases. That is protective measure from infection, but it means that optimizing and prioritizing mental health care is not always be possible*. (South Africa)

*The situation can be described as worrisome, and potentially perilous given the category of patients in question and the state of neglect attributable to this branch of medicine*. (Sierra Leone)

*The main need of the country is to allay anxiety of the masses by providing correct and legitimate information and involving public in the war against COVID*. (Zambia)

*People need support to deal with fears, stress, anxieties and distress of poverty, job/income loss as well as challenges of working at home in mostly inappropriate environments*. (Zimbabwe)

Mental health experts cited that mental health during this pandemic was not a priority. The already taxed resources will be put under additional strain due to the rise in mental health needs of the countries during this crisis.

…* no priority has been placed on issues of mental health during this crisis. It has never attracted much attention and chances are that the process of staff redistribution might affect the already understaffed solitary psychiatric Hospital* (Sierra Leone)

*Sadly mental health is an afterthought as the hierarchy in health limits the collaborative thinking for the good of all-every cadre feels and fronts their needs at the expense of self-care and aftercare management which is mental health-Within the mental health teams and across*. (Kenya)

*The already strained mental health care system will be under further strain, a general trail toward prioritizing COVID will come at a cost of mental health service provision. …. a large proportion of citizens, many of whom are in the informal labor market, now find themselves without an income which has a ripple effect on issues of compliance with medication as transport fees to health facilities may not be possible. The implications therefore are that the measures to protect society against COVID will invariably result in deterioration of other equally important health conditions including mental health conditions*. (South Africa)

*Mental health is a priority during this time, however given the limited number of mental health resources, people have been missing the opportunity to mental health information*. (Tanzania)

*Mental health is not a priority. The government is focusing on controlling transmission of the virus, protecting the economy, which is understandable, however, mental health could be mentioned more than under the 1% of the time it has come up in press meetings*. (Botswana)

*At first when we just started responding to COVID-19 as a country of course little or even attention was given to mental health to the point that all the mental units in the general hospitals were turned onto treatment units for COVID but as time went by and the government took some preventive measure including closing off borders and introducing lockdown, the mental health need started to be recognized as a results of a number of antisocial behaviors of violence and emotional outbursts in communities and among people hence the Ministry of health instituted psychosocial support subcommittee from all the response taskforce to focus at addressing the psychosocial needs*. (Uganda)

It was highlighted that some of the mental health needs required at this time are different from what is needed in a stable environment. Firstly, they felt that the mental well-being of individuals may be addressed by the clarity and consistent communication by governments, which may lead to some assurance that they are taking the necessary measures to deal with the pandemic in the various countries. They cited the need for improved access to avenues where individuals may seek mental health and psychosocial support, suggesting (1) there is need for more awareness on mental health needs that may arise because of the pandemic, (2) a need for clear and structured referral systems that will help in the management of mental health needs, and (3) a need for alternative methods of support such as the use of telemedicine or eMedicine to interact with persons in need of psychosocial support. Increased awareness and education for families of those already living with mental health disorders as these times may present adversity to the individuals possibly leading them to a worsened state.

*The major mental health needs would be around calmness in understanding the nature of the virus, providing health workers with sufficient information regarding their safety while interacting with “unknown clients” and providing continuous mental health debriefing for improved resilience. Providing linkage to appropriate services identified based on the need*. (Kenya)

*While the medical urgency of the pandemic needs to take priority, this needs to be balanced against the mental health needs that will be accentuated by or arise because of the pandemic. Our country is in a position where re-traumatisation seems inevitable. COVID-19 comes at a time when we have not yet recovered from the psychological impact of the HIV pandemic. Some of the reactions to COVID-19, such as stigmatization of the infected are the same as happened with the HIV pandemic*. (South Africa)

*The COVID-19, without need for much emphasis, could leave traces of mental instabilities ranging from PTSD, Depression, anxiety disorders and other psychological problems. In the light of these, mental health needs in the country may include psychosocial support, psychotherapists, Psychotropic drugs, support for mental health education nationwide*. (Sierra Leone)

*Individuals with mental illness may have heightened emotional responses and may therefore show more vulnerability to the stress associated with COVID-19, increasing the risk of relapses or worsening of existing conditions. The fear and worry about the threatened lives and livelihoods may result in individuals developing anxiety and depressive symptoms. Patients with serious mental illness and those with cognitive deficits may find it hard to understand and institute behavioral changes required of them such as hand washing and additional hygiene practices, the now mandatory practice of wearing of masks in public may not be understood and masks may feel induce feelings of claustrophobia, social distancing and being forced to stay at home may be an additional stressor. These factors may negatively impact disease containment measures or have an added burden to the careers*. (South Africa)

*Coping with fear, which can be mitigated by receiving the right information on COVID-19 from reliable sources*. (Tanzania)

*The main need of the country is to allay anxiety of the masses by providing correct and legitimate information and involving public in the war against COVID*. (Zambia)

*People need support to handle COVID-19 and Lockdown-related mental health issues such as fear, uncertainty, anxiety, and depression*. (Zimbabwe)

*Capacity building in terms of providing mental health services. Information sharing and awareness about the different types of mental health illness*. (Uganda)

Despite the lack of focus in mental health needs in the countries, some have placed a few measures in place to meet these needs. Some countries initiated training of mental health and lay providers in Psychological First Aid (PFA) to help manage the needs of healthcare workers, identified COVID cases, their families as well as the public.

*Community engagement (to a minimal extent) on the consequences of the pandemic on mental health*. (Sierra Leone)

*Trainings of the mental health providers in PFA and deployment to quarantine sites, hospital, and tele counseling*. (Kenya)

*While patients continue to receive treatment as usual, various support structures have been put in place to provide frontline workers with psychological support as the pandemic poses the risk of an added stressor to this group*. (South Africa)

*Establishment of the subcommittee of MHPSS in all the different taskforce teams responding to COVID. Social media engagement by different partner both from the NGO world and government*. (Uganda)

*Creation of a national psychosocial task force which has been tasked with developing mental health guidelines…establishment of a toll free number where individuals may call to get assistance on psychosocial matters…social support such as provision of food baskets during lockdown*. (Botswana)

It was also identified that the provision of mental health care/services may be hindered during this period due to various reasons: inadequate policies, limited human resource trained in mental health care and where to access these services, movement restrictions. Fear of contracting the virus may also hinder help-seeking behavior as well as the general stigma associated with mental health. It was also noted that there was a lack of collaboration and coordination of potential stakeholders who would help mitigate mental health needs.

*Lack of knowledge among the populace on mental health and where to access these services. Stigma and discrimination associated with mental health that still needs to be debunked* (Kenya)

*The financial and economic barriers already stated. Social distancing means that physical consultations with mental health care providers has to be limited as much as possible. Users who are stable are allowed to have digital/electronic consultations with their providers. This useful resource is available to a very small proportion of the population as a greater number of the population has no access to gadgets that would allow such use, and no access to affordable data*. (South Africa)

*Resources are mainly directed to frontline staff and the vulnerable groups are at risk of being forgotten*. (Zimbabwe)

*People are afraid to come to hospitals fearing contracting the virus. Lack of enough PPE to healthcare workers making them prone to contracting the virus as well*. (Tanzania)

*Lack of coordination and communication, people do not know where to go for help*. (Botswana)

*Lack of leadership, lack of collaboration between various health professionals, lack of Infrastructure. Lack of transparency in extending rightful information, lack of regulatory measures in strengthening the basic safety measures across the country*. (Zambia)

*The processes of developing mental health support protocols and actual service delivery are very bureaucratic. There is a lot of going back and forth especially with NGOs such that the service takes forever to kick off. This has resulted in catastrophic incidences, including suicide, incest, and development of severe mental disorders*. (Zimbabwe)

*Resources in terms of information and finances. Attitude of people toward mental health services. Few numbers of mental health experts. Poor policies*. (Uganda)

As mentioned above, some countries in SSA have released guidelines to help manage the mental health and psychosocial needs of its society. Interviewed practitioners varied in their view of the appropriateness of the guidelines, which some found to be appropriate while others found them lacking to meet the needs of the community during this pandemic.

*The plan is lacking on the coordination mechanism being that we interpret distress and abnormal behavior differently in Africa due to the stigma therefore healthcare seeking habits are limited and biased to certain populations. Vulnerable populations such as children, people with disabilities, the elderly are not given much considered. Conversely the adolescents, youth and general population have a low risk perception for mental health and might therefore miss out on timely referrals. We might therefore need to address mental health preparedness with the same urgency and approach we do with gender based violence or emergency delivery-develop a clear referral pathway and coordination standard operation protocol and procedure, sensitize both community and frontline health workers on the cascade and chain of management and offer collaborative support to other peers who might not be brave enough to shout out for help*. (Kenya)

*Yes, they are adequate. The SOPs released by the national government specifically encourages the identification of the specific groups and guidelines on how to handle each of them*. (Kenya)

*The initiative of developing guidelines in response to COVID is a good one. An important main objective of the guide is the fact that mental health care standards need not be compromised because of the pandemic, and mental health care users should continue receiving good quality care and continued screening for common mental conditions at primary health care has to continue. However, it needs to be taken into cognizance that the guideline requires added resources, such as additional patient screening for COVID, isolation of patients with suspected COVID. Not all facilities will have resources for such necessary measures as the system is already operating at or above capacity. The guidelines correctly stipulate that mental care should be given where it is due and where patients are infected with COVID, support must be given to patient family. The only vulnerable populations addressed by the guide is children, there is not special guide toward dealing with other vulnerable population*. (South Africa)

*(they need) to strengthen mental health services and involve mental health professionals in the management of every patient with COVID*. (Zambia)

*Mental health was not initially part of the COVID-protocol from the onset. There was need to integrate it from the beginning e.g., at pre-test stage*. (Zimbabwe)

## Discussion

The countries differed from each other in terms of the occurrence of the first case, number of confirmed cases, rates of mortality and measures adopted. Mental health guidelines for COVID-19 were identified in 5 countries. Most mental health guidelines were developed based on the World Health Organization considerations on how to protect your mental health released in March 2020 (13). Our findings showed that the countries with mental health guidelines for use during this pandemic, had clearly stated purposes and aims. In addition, South Africa clearly identified mental health services as essential during this time as indicated in section 27 (2) of the disaster management act 2002 (24). This is supported by Secretary-General António Guterres of the United Nations who stated, “*Mental health services are an essential part of all government responses to COVID-19. They must be expanded and fully funded”* (25). Kenya went beyond what was expected and developed standard operating procedures (SOPS) for counselors and psychologists providing MHPSS services in the midst of this crisis (20).

SSA countries have shown economic growth at varying rates since the 1990's, and some have alluded the growth rates to be related to the quality of policies and institutions in the countries (26). In our study, we found no link between countries of high rates of economic growth and the presence of mental health policies.

It is important to note that, mental health in SSA has been given less priority during this pandemic and received little attention from the governments. This finding is however, to be expected as mental health policies have generally not been priority in most SSA countries (27, 28). For example, by 2020, the Nigerian Mental Health Services Delivery Policy had yet to be effected into law (28); In Zimbabwe, the policy had been last reviewed in 1996 (27). The focus, therefore, is more on prevention of COVID-19 infection and physical symptoms. The blind spot on mental health in policy reforms is evidenced by lack of mental health guidelines in Botswana, Sierra Leone, Ethiopia, Zambia, and Zimbabwe which shows that mental health is not at the frontline of health regulations and agendas. Our results are comparable with literature which observed that mental health remains under prioritized in Africa (29, 30) with majority of African countries lacking mental health policies or having outdated mental health legislature (31). In addition, COVID-19 budgets focused less on mental health but more on securing protective clothing and testing kits. Jacob et al. (32) agree that 70% of countries in Africa spend < 1% of the total health budget on mental health. Given the context of low overall funding for mental health, there is an urgent need for governments to increase funding for mental health in Africa. Even without the influence of a pandemic such as COVID-19, the mental health system in SSA is already taxed and insufficient with challenges related to the economic and development inequalities as well as social and cultural contexts (30).

The guidelines reviewed underscore the need to put appropriate measures in place that guide the provision of mental health and psychosocial support during and after the outbreak. They highlight that it is not only individuals, families directly affected or health care workers who are prone to psychological deterioration during and after the COVID-19 outbreak, but the whole society. The Inter-Agency Standing Committee (33) uses MHPSS to unite a broad range of actors responding to emergencies such as this one, underscoring the need for diverse, complementary approaches in the provision of appropriate support. It goes further to suggest that there are overarching principles to MHPSS response. These suggestions are echoed by Monteiro (30) who says that there is a lack of priority on mental health, as evidence not only in a lack of mental health policy, but most recently guidelines for the management of mental health issues that arise due to the COVID-19 crisis. Limited resources (both human and financial) for mental health leave our societies vulnerable. At 1.4 per 100,000, Africa compares unfavorably to the 9.0 per 100,000 mental health care workers reported globally (34). The professionals cited the need for not only improved access to care, as well as a need for clearly defined referral systems that cater to MHPSS needs during this time. These are systems that already need to be in place and bolstered during a crisis such as COVID-19, but this is not the case in many SSA countries.

Both the Kenyan and the Cameroon guidelines promote Psychological First Aid (PFA). According to WHO, (35) PFA is a humane, supportive, and practical assistance to fellow human being who have recently suffered exposure to serious stressors. During COVID-19, PFA is a non-professional framework that works to provide comfort and practical support, focusing on mental and psychosocial response during and after a crisis. In situations such as these, literature has shown that the number of people whose mental health is affected tends to be greater than the number of people affected by the disease (36). The health implications of contracting COVID-19 compounded by the stringent measures taken by governments to curb the spread of disease heighten risk for mental health issues. A review by Brooks et al. (37) suggests that individuals quarantined or in isolation require attention as they are deprived of their freedom. For individuals already suffering mental health problems, da Silva et al. (38) highlights that the strict measures in place make them more vulnerable to environmental stressors. Such stressors include long/extended quarantine duration, fears of infection, frustration, boredom, inadequate supplies, inadequate information, financial loss, and stigma. They suggest that the negative psychological effects may lead to post-traumatic stress symptoms, confusion, and anger. During this period children are also exposed to large amounts of information and high levels of stress and anxiety from the adults around them (39); therefore, there is need for the developed guidelines to address different population groups and their needs.

Victor and Ahmed (40) suggest that epidemics of the developing world differ from epidemics of the developed countries, both in causing agents and in their proportions. With isolation, reduced opportunity for leisure activities, increased financial stress as a consequence of reduced economic activity; mental health consequences of the pandemic are inescapable (41). Particularly in SSA as COVID-19 is an added burden in conditions where these factors are pre-existing. The Dohrenwend model describes how mental health relates to socio-economic stress and suggests two things. First, that social causation—or the financial strain, the increased exposure to violence and the food insecurity linked to poverty, combined with reduced access to social safety nets such as food banks, income support or family or friends who can offer loans—all increase the likelihood of people developing mental-health problems. Second, that those with pre-existing mental health conditions are most at risk of “social drift” —in which mental illness increases a person's exposure to economic shocks and spurs their fall into poverty (42). The loss of employment opportunities, reduced pay, together with lockdowns and movement restrictions have influenced deterioration of the social and economic conditions of many. Many are at risk for a decline in their mental health thus highlighting the need to address the social and economic conditions that contribute to poor mental health during this time (43).

While the odds may be against SSA with regards to mental health and COVID-19, the emergence of telemedicine in countries such as Nigeria and South Africa show promise in for future clinical care with reduced physical contact and therefore COVID-19 risk reduction (44, 45).

### Recommendations

From our findings we recommend that governments need to put the appropriate measures in place to mitigate the mental health effects related to COVID-19. There is also need for clearly defined referral pathways that cater to MHPSS needs during this time. The systems that already in place, need to be bolstered during this crisis. There is need for guidelines that address specific vulnerable populations (children, adolescents, geriatric, differently abled, etc.) and their needs. The needs of such populations vary from that of general population and require more specialized support.

### Strengths and Limitations

A strength of our study is the availability of COVID related MHPSS guidelines from included countries to critique. The inclusion of mental health experts working within these countries to give their views on the effects of the pandemic on the mental health of the African population. The limitations of this study are that it was self-interview soliciting expert opinion, which is lower in the hierarchy of evidence. However, expert opinion has been found to be an acceptable means of providing evidence as it is based on factual information and brings new information particularly in new or emergent areas (46). SSA countries are not homogenous and have various factors driving the socioeconomic landscape and policy, this poses a limitation to having a blanket assessment of the situation but, similarities such as rapid urbanization (47) make it prudent to assess these countries together.

## Conclusion

We note that a number of SSA countries overlooked the development of MHPSS guidelines at a critical time where the already unmet mental healthcare needs were anticipated to be under further strain. The development of MHPSS guidelines in the wake of a pandemic cannot be overstated. The consequences of added pressure because of COVID-19 to the mental health care system and simultaneous the allocation of resources away from these services will have a lasting effect for years to come require careful consideration. We would like to propose that the pandemic may be an opportunity to strengthen and improve on mental healthcare policies, as a first step by governments toward improved mental health care provision, particularly for under resourced settings.

## Author Contributions

KM, OM, and GNW reviewed the various guidelines and responses from the online qualitative survey. GNW interpreted the data. KM, OM, GNW, and VN developed the manuscript, critically revised it, and approved the final manuscript for submission. All authors contributed to the development of the project.

## Conflict of Interest

The authors declare that the research was conducted in the absence of any commercial or financial relationships that could be construed as a potential conflict of interest.

## Abbreviations

COVID-19: Severe Acute Respiratory Syndrome Coronavirus 2
MHPSS: Mental Health and Psychosocial Support
MHPAF: Mental Health Preparedness and Action Framework
MHSS: Mental Health Surveillance System
PFA: Psychological First Aid
PTSD: Post Traumatic Stress Disorder
SSA: Sub-Saharan Africa
WHO: World Health Organization.
